# Data Flush

**DOI:** 10.1162/99608f92.681fe3bd

**Published:** 2022-05-09

**Authors:** Xiaotong Shen, Xuan Bi, Rex Shen

**Affiliations:** † School of Statistics, University of Minnesota, Minneapolis, MN 55455; ‡ Carlson School of Management, University of Minnesota, Minneapolis, MN 55455; ⋄ Department of Statistics, Stanford University, Palo Alto, CA 94305

**Keywords:** Census, differential privacy, distribution preservation, data integration, statistical inference

## Abstract

Data perturbation is a technique for generating synthetic data by adding “noise” to raw data, which has an array of applications in science and engineering, primarily in data security and privacy. One challenge for data perturbation is that it usually produces synthetic data resulting in information loss at the expense of privacy protection. The information loss, in turn, renders the accuracy loss for any statistical or machine learning method based on the synthetic data, weakening downstream analysis and deteriorating in machine learning. In this article, we introduce and advocate a fundamental principle of data perturbation, which requires the preservation of the distribution of raw data. To achieve this, we propose a new scheme, named *data flush*, which ascertains the validity of the downstream analysis and maintains the predictive accuracy of a learning task. It perturbs data nonlinearly while accommodating the requirement of strict privacy protection, for instance, differential privacy. We highlight multiple facets of data flush through examples.

## Media Summary

The explosive growth of large volumes of data with complex structures demands the wide usage of data in applied sciences. In privacy protection, data perturbation is an effective technique. For instance, it privatizes the U.S. Decennial Census Data to protect the confidentiality of individuals by the standard of differential privacy Kenny et al. (2021) and United States Census Bureau (2020). However, the scientific community criticizes such privatization methods for producing synthetic data invalidating downstream statistical analysis at the expense of satisfying differential privacy. The lack of statistical accuracy raises concern for the interpretability and reliability of any statistical and machine learning solutions to a practical problem. Despite its great potential in domain sciences, the data science community underappreciates the data perturbation technique. Here, we introduce and advocate a fundamental principle of data perturbation that retains the distributional information, validating downstream analysis, and delivering accurate prediction and reliable interpretation, for raw and privatized data.

## 1. Introduction

Data perturbation gives rise to synthetic data by adding noise to raw data, which has had vast applications since the pioneering work of Breiman on estimating the prediction error in regression (Breiman, 1992). In the data privacy domain, data perturbation can ensure a prescribed level of privacy protection by imposing a suitable noise level (Day One Staff, 2018; Dwork, 2006; Erlingsson et al., 2014; Kaissis et al., 2020; Santos-Lozada et al., 2020; Venkatramanan et al., 2021). In statistics and data science, data perturbation is an effective tool for replicating a sample, for example, developing Monte Carlo methods of model selection (Breiman, 1992; Shen & Ye, 2002). In this situation, data perturbation generates synthetic data to resemble raw data in terms of distribution. Despite its great potential in many domain sciences, the data science community underappreciates the data perturbation technique.

In the differential privacy literature, data perturbation privatizes raw data to satisfy the requirement of *ε*-differential privacy (Dwork, 2006; Dwork, McSherry, et al., 2006), for example, by the Laplace method (Dwork, McSherry, et al., 2006; Dwork & Roth, 2014). Data perturbation can also mask sensitive classification rules in data mining (Delis et al., 2010). One major challenge for privacy protection is that most privatization methods suffer from information loss in a privatization process to satisfy a prescribed level of privacy protection (Gong & Meng, 2020; Goroff, 2015; Santos-Lozada et al., 2020). As a result, privatization weakens downstream statistical analysis and yields unreliable machine learning solutions. One remedy to information loss is to lower the level of protection to trade for reasonably good accuracy of statistical analysis. This common practice refers to as low-error-high-privacy differential privacy in the survey literature (Chen et al., 2016; Reiter, 2019).

In the statistics literature, data perturbation has been utilized for model assessment as in the generalized degrees of freedom (Ye, 1998) and for developing adaptive model selection criteria (Shen & Huang, 2006; Shen & Ye, 2002) and model averaging criteria for nonlinear models (Shen et al., 2004), estimating the generalization error (Shen & Wang, 2006), and performing causal inference (Xue et al., 2021). One challenge here is how to generate synthetic data to validate statistical inference despite the significant progress for statistical prediction.

In many applied sciences, synthetic data must meet task-specific requirements for an end-user. In privacy protection, synthetic data or privatized data must meet some privacy protection standards to guard against disclosure. In statistics, synthetic data replicates a random sample so that users can perform statistical analysis, simulate phenomena and operational behaviors of a real-world process, and train machine learning algorithms. For instance, Candes et al., 2018 uses knockoffs, a special kind of synthetic data, to estimate the Type I error or false discovery error rate in feature selection. In such a situation, one challenge is how to ensure that synthetic data would represent raw data while satisfying task-specific requirements to meet an end user’s needs.

To meet the challenges, we first review the data perturbation technique and introduce a scheme of data perturbation, what we call *data flush*, to guide users to design a perturbation process to validate the downstream analysis and yield reliable solutions. Then, we demonstrate the utility of data flush in two disparate yet intertwined areas: statistical inference and differential privacy. Critically, this scheme enables to satisfy any level of privacy protection for differential privacy while maintaining the statistical accuracy of privatized data as if one used raw data. Finally, we showcase the data-flush scheme in that it can simultaneously satisfy requirements in both differential privacy and statistical inference.

The data-flush scheme is distinctive in three ways. First, it generates multiple perturbed copies of the raw data following a target distribution. Second, it can ensure differential privacy while preserving the target distribution. Third, it applies to nearly all kinds of data, particularly continuous, discrete, mixed, categorical, and multivariate. To the best of our knowledge, Bi and Shen, 2021 and Woodcock and Benedetto, 2009 are only methods of preserving a target distribution, where the former satisfies differential privacy while the latter only limits disclosure risk. Furthermore, data flush also maintains its link with the raw data identifier or the user’s identification, permitting data integration, data sharing, and personalization.

This article consists of five sections. Section 2 introduces the data-flush scheme and discusses its applicability in differential privacy and statistics. Section 3 develops a pivotal inference method based on data flush, which ascertains the validity of statistical inference. Section 4 applies the data-flush scheme to the 2019 American Community Survey Data to demonstrate its effectiveness in differential privacy protection and contrast statistical inference before and after privatization. Section 5 discusses future directions of data perturbation. The Appendix contains some technical details.

## 2. Data flush

This section introduces a fundamental principle of data perturbation, stating that data perturbation must preserve the distribution of raw data to ascertain the validity of the downstream analysis and the reliability of a machine learning solution. Applying this principle, we derive a data perturbation scheme, called data flush, based on a family of nonlinear data perturbations, which simultaneously satisfy the requirements of differential privacy and valid statistical analysis.

### 2.1. Data perturbation.

Data perturbation adds noise directly to raw data (Breiman, 1992; Shen & Ye, 2002; Ye, 1998), which is called linear perturbation. As argued in Bi and Shen, 2021, a nonlinear perturbation is necessary to preserve data distributions while satisfying the requirement of *ε*-differential privacy (Dwork, 2006; Dwork, McSherry, et al., 2006).

Next, we suggest a data-flush scheme, permitting more flexibility beyond linear perturbation for various types of data.

#### Univariate continuous distributions.

Given an independent sample (*Z*_1_, . . .,*Z*_*n*_) from a cumulative distribution function (CDF) *F*, we perturb the raw sample to follow a prespecified target distribution *R*. For example, *R* can be a standard normal distribution or a uniform distribution. But more commonly, *R* = *F* if *F* is known and $R=\hat{F}$ otherwise, where $\hat{F}$ is a smooth estimate of the empirical CDF (Bi & Shen, 2021) or a model-specific distribution function (Reiter, 2005) such as a normal distribution with an estimated mean.

First, we sample (*U*_1_, · · ·,*U*_*n*_) from Uniform[0, 1] and relabel them so that the rank of *U*_*i*_ in (*U*_1_, · · ·,*U*_*n*_) remains the same as that of *Z*_*i*_ in (*Z*_1_, . . .,*Z*_*n*_). This transformation from *Z*_*i*_ to *U*_*i*_ encodes a positive (Spearman’s rank) correlation between the perturbed and the original samples, c.f., Lemma 1. Second, suppose we are interested in generating *m* perturbed samples. We add independent continuous noise *e*_*ij*_, *j* = 1, . . .,*m*, to *U*_*i*_ independently. Then, we map *U*_*i*_ + *e*_*ij*_ to yield a perturbed sample following the target distribution *R*:

(2.1) $$Z_{ij}^{*}=H\left(U_{i}+e_{ij}\right),\;H(\cdot )=R^{-1}(G(\cdot ));\;i=1,\ldots ,n,\;j=1,\ldots ,m,$$

where *G* is the CDF of *U*_*i*_ + *e*_*ij*_.

The perturbed observation $Z_{ij}^{*}$ follows the target distribution *R* while $Z_{1j}^{*},\ldots ,Z_{nj}^{*}$ are independent across *i* = 1, . . .,*n*. The distribution of *e*_*ij*_ can be chosen to satisfy a task-specific requirement.

#### Multivariate continuous distributions.

Given an independent sample (***Z***_1_, . . .,***Z***_*n*_) following a *p*-dimensional continuous distribution *F*, we apply (2.1) to each component $Z_{i}^{\left(j\right)}$ through the probability chain rule, where $Z_{i}=\left(Z_{i}^{\left(1\right)},\ldots ,Z_{i}^{\left(p\right)}\right)$. That is, $Z_{i}^{\left(1\right)}$ yields $Z_{ij}^{(1)*}$, then $Z_{i}^{\left(2\right)}$ given $Z_{ij}^{(1)*}$ yields $Z_{ij}^{(2)*}$ as in (2.1), and so forth. A perturbed sample is

(2.2) $$Z_{ij}^{(1)*}=H^{\left(1\right)}\left(U_{i}^{\left(1\right)}+e_{ij}^{\left(1\right)}\right),Z_{ij}^{(l)*}=H^{\left(l\right)}\left(U_{i}^{\left(l\right)}+e_{ij}^{\left(l\right)}\right);\;j=1,\ldots ,m,\;l=2,\ldots ,p,$$

where $\left(U_{1}^{\left(l\right)},\cdots ,U_{n}^{\left(l\right)}\right)$ is a Uniform[0, 1] random sample for $Z_{i}^{\left(l\right)}$ and $H_{i}^{\left(l\right)}={\left(R_{i}^{\left(l\right)}\right)}^{-1}(G(\cdot ))$ applies to $Z_{i}^{\left(l\right)}$ given $Z_{ij}^{(1)*},\ldots ,Z_{ij}^{(l-1)*}$ as in (2.1), with *R*^(*l*)^ the conditional distribution of $Z_{i}^{\left(l\right)}$ given $Z_{ij}^{(1)*},\ldots ,Z_{ij}^{(l-1)*}$. Note that is unnecessary to relabel $\left(U_{1}^{\left(l\right)},\cdots ,U_{n}^{\left(l\right)}\right)$, *l* = 2, . . .,*p*, as the first variable in the chain rule has preserved the identifier of raw data.

#### Discrete and mixed distributions.

A generalization of (2.2) to discrete or mixed distributions, including the empirical distribution, is achieved through a smooth version of noncontinuous *F*, which agrees with *F* at its jump values, see Bi and Shen, 2021 for more details. Then, (2.2) applies by replacing *F* with its smooth version.

### 2.2. Key properties and benefits.

Several characteristics of data-flush in (2.2) are worth mentioning. First, $Z_{ij}^{*}$ follows the target distribution *R*. This distribution-preservation property ensures statistically valid analysis on perturbed data. Second, $Z_{ij}^{*}$ is positively correlated with $Z_{i}^{\left(1\right)}$, as measured by the Spearman’s rank coefficient when *e*_*ij*_ is small; *i* = 1, . . .,*n*; c.f., Lemma 1. In contrast to synthetic data generation methods, this property guarantees that data flush maintains the data identifier or index *i* between $Z_{ij}^{*}$ and ***Z***_*i*_, which is accomplished through the first variable of interest $Z_{i}^{\left(1\right)}$. Hence, it permits personalized analysis at the individual level. Third, $\left(Z_{i1}^{*},\ldots ,Z_{im}^{*}\right)$ are conditionally independent given $U_{i}=\left(U_{i}^{\left(1\right)},\ldots ,U_{i}^{\left(p\right)}\right)$; *i* = 1, . . ., *n*, while $\left(Z_{1j}^{*},\ldots ,Z_{nj}^{*}\right)$ are unconditionally independent; *j* = 1, · · ·,*m*.

#### Lemma 1.

*In* (2.2), *the Spearman’s rank coefficient* $\rho \left({\left\{Z_{i}^{\left(1\right)}\right\}}_{i=1}^{n},\;{\left\{Z_{ij}^{(1)*}\right\}}_{i=1}^{n}\right)\to 1$ *as e*_*ij*_ → 0 *in probability; i* = 1, . . .,*n*, *j* = 1, . . .,*m.*

The proof is given in the Appendix.

### 2.3. Applications.

#### 2.3.1. Differential privacy.

This subsection reviews the application of data perturbation in differential privacy and present the advantages of data flush. Differential privacy becomes the gold standard of privacy protection for publicly released data, for example, census data (Kenny et al., 2021; United States Census Bureau, 2020). Given a prescribed level (i.e., privacy factor) *ε* > 0 of privacy protection, *ε*-differential privacy (Dwork, 2006) requires that the alteration of any original data leads to a small change of the released information.

The differential privacy literature focuses on the design of privatization methods satisfying *ε*-differential privacy. Towards this end, Wasserman and Zhou, 2010 laid the statistical foundation of differential privacy. As noted in Goroff, 2015,Santos-Lozada et al., 2020, and Gong and Meng, 2020, essentially all privatization methods weaken downstream statistical analysis at the expense of achieving a prescribed level of privacy protection, which is referred to as the trade-off between data privacy and usefulness. Moreover, differential privacy usually entails an impractical requirement on raw data, namely, the bounded support of its underlying data distribution (Wasserman & Zhou, 2010).

To alleviate the accuracy loss and the boundedness requirement, scientists attempt to approximately preserve some summary statistics of raw data in a privatization process. Snoke and Slavković, 2018 suggested a privatization method by maximizing a distributional similarity between privatized and raw data. Liu, Vietri, Steinke, et al., 2021 (i.e., PMW) leveraged public data as prior knowledge to improve differentially private query release, and Liu, Vietri, and Wu, 2021 (i.e., GEM) developed an iterative method to approximately preserve the answers to a large number of queries for discrete data. Boedihardjo et al., 2021 improved the statistical accuracy of the Laplacian method by estimating the distribution of raw data. However, none of these methods preserved the probability distribution of raw data, although they intend to retain some summary statistics such as the distributional similarity and answers of queries. Furthermore, GEM focused on a weaker version of *ε*-differential privacy, known as (*ε*, *δ*)-differential privacy (Dwork, Kenthapadi, et al., 2006), where *δ* denotes the probability of information being leaked.

Despite the progress, information loss for downstream statistical analysis prevails for most privatization methods. Preservation of summary statistics may be inadequate as an evaluation metric requires the knowledge of the data distribution for statistical analysis or a machine-learning task. For example, GEM suffers from a loss of statistical accuracy even if it intends to preserve the discrete distribution of multi-way interactions. As illustrated in Table 1, GEM not only renders a significant amount of accuracy loss in terms of predictive performance and parameter estimation in regression analysis but also requires excessive computation to achieve privatization. In contrast, the data-flush scheme (2.2) maintains high statistical accuracy due to distribution preservation, which has greater data usefulness for downstream analysis. More simulation details are provided in the Appendix.

Data flush adds suitable noise to guarantee a prescribed level of privacy protection while applying a nonlinear transformation to preserve a target distribution to validate the downstream analysis and provide reliable solutions. For example, one can adopt a version of (2.2) with noise *e*_*ij*_ following a *Laplace*(0, 1*/ε*) distribution to guarantee *ε*-differential privacy (Bi & Shen, 2021), and a smoothed empirical CDF to approximates the original data distribution. However, the empirical CDF has to be built upon an independent sample to satisfy the definition of *ε*-differential privacy. Public data from similar studies can serve as the independent sample, such as past American Community Survey data for the current American Community Survey or Census. As an alternative, one can also consider a holdout sample, which is a random subset of the raw data (Bi & Shen, 2021). In this situation, the holdout sample is fixed once selected. Any alteration, query, or release of the holdout sample is not permissible. This guarantees the strict privacy protection of individuals in the holdout sample. In this sense, differential privacy does not apply to the holdout sample, since query and alteration as required by the definition of differential privacy are not allowed.

#### 2.3.2. Inference.

This subsection briefly comments on data flush as a tool for statistical inference. A crucial aspect of data flush is its capability of recovering the exact distribution of a pivotal quantity in the finite sample regime, as shown in Theorem 1. In contrast, a resampling method such as bootstrap (Efron, 1992; R. J. Tibshirani & Efron, 1993) approximates the distribution of a pivotal via a Monte Carlo method, which can not recover the exact distribution in the finite sample regime. Moreover, data flush has the great potential to treat the issue of the bias in inference after model selection, as demonstrated in Section 3. In contrast, standard bootstrap suffers from the difficulty of discontinuities of an estimate (Efron, 2004).

#### 2.3.3. Other applications.

Data flush has applications in other areas.

##### Model sensitivity.

To quantify the impact of model selection on estimation, Ye, 1998, Shen and Ye, 2002, and Shen and Wang, 2006 define the generalized degrees of freedom using the notion of model sensitivity through a linear perturbation form $Z_{i}^{*}=Z_{i}+\varepsilon_{i}$ with *ε*_*i*_ ~ *N*(0, *ε*^2^) for a Gaussian sample (*Z*_1_, . . .,*Z*_*n*_). Data flush provides a means of evaluating the model sensitivity for various data.

##### Data integration and personalization.

Data-flush in (2.2) retains a positive rank correlation between perturbed and raw observations for the first component $\left(Z_{1}^{\left(1\right)},\cdots ,Z_{n}^{\left(1\right)}\right)$, as suggested by Lemma 1. This first component serves as a data identifier for data integration and personalization. In privacy protection, for instance, privatized data is released for one time period and can be merged with forthcoming data for different periods via a data identifier. By comparison, a resampling method distorts any data identifier.

## 3. Pivotal inference

This section develops a data perturbation tool for pivotal inference based on raw data without privacy concerns. We apply the data-flush scheme (2.2). The perturbed data replicate raw data to simulate the sampling distribution of a pivotal, which constructs a confidence interval or a test for parameter *θ*.

Let $T=T(\theta ,\;\hat{\theta })$ and $\hat{\theta }=\hat{\theta }(Z)$ denote a pivotal and an estimate based on a random sample ***Z*** = (*Z*_1_, . . .,*Z*_*n*_), with each *Z*_*i*_ following a probability distribution *F*(*θ*), and *F* is known but *θ* is unknown. The distribution of *T* is independent of *θ*, which requires a Monte-Carlo resampling method such as bootstrap to estimate, as its analytic form is often unavailable. However, such a resampling method may suffer the difficulty of inference after model selection. As pointed out in Efron, 2014, one needs to adjust for bootstrap by smoothing through bagging (Breiman, 1996) to treat the erratic discontinuities of an estimate. In such a situation, data flush provides an effective means of approximating the distribution of *T*.

Data flush generates a pseudo sample $Z^{*}=\left(Z_{1}^{*},\ldots ,Z_{n}^{*}\right)$ from *Z* = (*Z*_1_, . . .,*Z*_*n*_) according to (2.2) so that the conditional distribution $Z_{i}^{*}$ given ***Z***_*i*_ follows a target distribution $R={F(\theta )|}_{\theta =\hat{\theta }}$. Then, we compute the perturbed pivotal $T^{*}=T\left(\hat{\theta },\;{\hat{\theta }}^{*}\right)$, where ${\hat{\theta }}^{*}=\hat{\theta }\left(Z^{*}\right)$ is the estimate based on *Z*^∗^ by applying the same statistical procedure for $\hat{\theta }\left(Z\right)$.

Theorem 1 exhibits a useful yet less known fact about the conditional distribution of *T*^∗^ given ***Z***, which can substitute an unknown distribution of *T* for pivotal inference. Note that the former can be computed but not the latter.

### Theorem 1.

*(Distribution preservation) The conditional distribution of T*^∗^ *given* ***Z*** *remains the same as the distribution of T for any* ***Z****. Hence*, *any test or a confidence interval on the conditional distribution of T*^∗^ *given* ***Z*** *is exactly as if the distribution of T would have been used.*

The proof is given in the Appendix.

#### Data-flush Monte-Carlo inference.

For an exact or asymptotic pivotal, we may compute the conditional distribution of *T*^∗^ given ***Z*** via a Monte-Carlo approximation while correcting bias through data perturbation to improve the finite-sample performance. Data perturbation permits estimation of the bias of a statistical procedure through repeated experiments as in simulations, as illustrated in a subsequent data example. The following data-flush Monte-Carlo method summarizes this proposal.

#### Step 1: Monte-Carlo approximation of the distribution of *T*.

Generate *D* independent perturbed samples $Z_{d}^{*}=\left(Z_{1d}^{*},\ldots ,Z_{nd}^{*}\right)$ according to (2.2), with each $Z_{1d}^{*}$ following $R=F(\hat{\theta })$; *d* = 1, . . .,*D*, *m* = *D*. Note that we may choose any continuous unbounded distribution of *e*_*ij*_ in (2.2) for a task-specific purpose (such as a Laplace distribution to satisfy *ε*-differential privacy). In what follows, *D* refers to as a Monte-Carlo size. Compute the perturbed pivotal $T_{d}^{*}=T\left(\hat{\theta },\hat{\theta }\left(Z_{d}^{*}\right)\right)$; *d* = 1, . . .,*D*. Compute the empirical distribution of $T_{1}^{*},\ldots ,T_{D}^{*}$, rendering the exact distribution of *T* as *D* → ∞.

#### Step 2: Bias-correction.

Compute the bias estimate $\hat{B}=D^{-1}\sum_{d=1}^{D}\left(\hat{\theta }\left(Z_{d}^{*}\right)-\hat{\theta }\right)$. Compute the biased-corrected estimate ${\hat{\theta }}^{c}=\hat{\theta }+\hat{B}$.

#### Step 3: Inference.

Use $T\left({\hat{\theta }}^{c},\theta \right)$ to construct a confidence interval based on the empirical distribution of $T_{1}^{*},\ldots ,T_{D}^{*}$.

Next, we illustrate this data-flush inference method by two examples.

#### Exact distribution of a pivotal.

The first example concerns the distribution of a pivotal quantity for construction of a confidence interval of the population mean *θ* of a normal distribution with unknown *σ*^2^. The pivotal is of the form $T(\bar{Y},\theta )=\frac{\bar{Y}-\theta }{S}$, where $\bar{Y}$ is the sample mean and *S* is the sample standard deviation. Here, we apply the data-flush inference scheme to simulate the distribution of perturbed pivotal *T*^∗^ and compare it with the bootstrapped pivotal (Efron, 1992) and the exact distribution of *T*. To generate perturbed samples for inference, we apply (2.1) with *e*_*ij*_ following a *Laplace*(0, 1*/ε*) distribution with *ε* = 0.01 and *R* being the CDF of $N\left(\bar{Y},S^{2}\right)$ given ***Z***.

**Figure 1.**
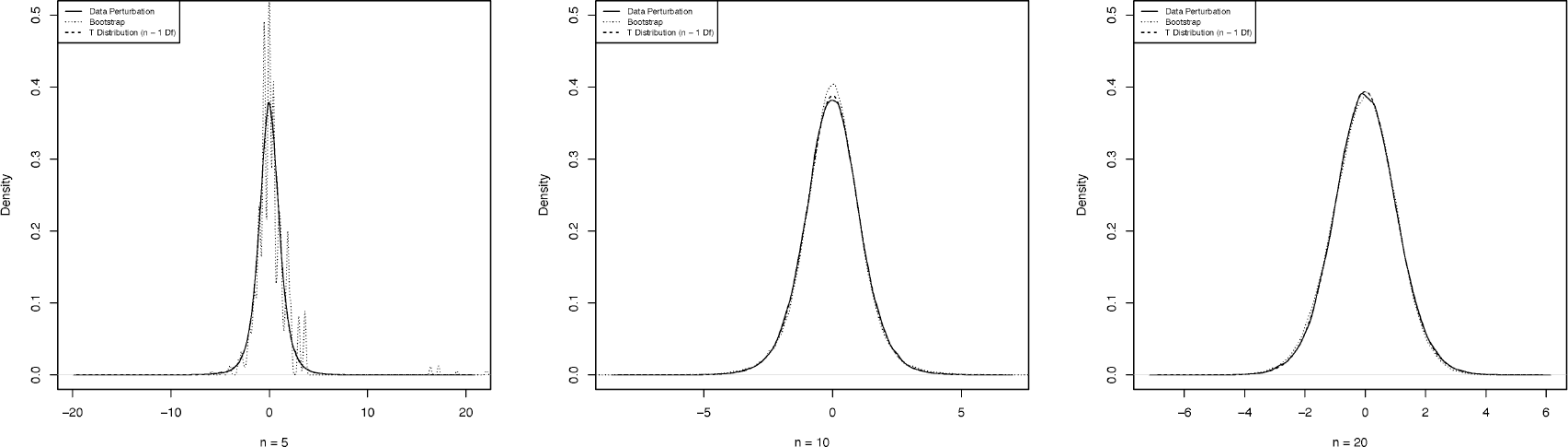
Illustration of the exact distribution of pivotal for three sample sizes *n* = 5, 10, 20 based on simulated data. Pivotal’s densities for data flush with a Monte Carlo size 10^5^, nonparametric bootstrap with a bootstrap size 10^5^, and the t-distribution on *n* − 1 degrees of freedom are represented by solid, dot, and dash curves, respectively.

Figure 1 reveals one salient aspect of data flush: It renders a nearly identical distribution of *T*, whereas nonparametric bootstrap differs substantially for a small sample size *n* = 5. In other words, nonparametric bootstrap’s approximation accuracy depends highly on the sample size *n*. Indeed, data flush yields an exact distribution of a pivotal as the Monte-Carlo size *D* → ∞. This observation agrees with the result of Theorem 1.

#### High-dimensional regression.

Our second example focuses on the construction of a confidence interval in linear regression on a vector of *p* predictors:

(3.1) $$Y_{i}=\beta^{T}X_{i}+\varepsilon_{i};\;\varepsilon_{i}\sim N\left(0,\sigma^{2}\right);i=1,\ldots ,n,$$

where *p* could be substantially larger than the sample size *n*, *β* = (*β*_1_, . . .,*β*_*p*_) is a vector of regression coefficients, ***X***_*i*_ = (*X*_*i*1_, . . .,*X*_*ip*_) ~ *N*(**0**,**Σ**) is a vector of predictors that are independent of the error *ε*_*i*_, and the (*j*, *k*)-th element of the covariance matrix **Σ** is *ρ*^|*j*−*k*|^, and *σ*^2^ is an unknown error variance. Our goal is to construct a confidence interval for an individual coefficient *β*_*l*_ with other covariates involving model selection.

In a high-dimensional situation, one often applies the method of regularization for dimension reduction. As a result of the inherent bias from regularization, a standard method needs debiasing and uses an asymptotic distribution of debiased LASSO estimate (Zhang & Zhang, 2014) with *L*_1_-penalty (R. Tibshirani, 1996) given a prespecified regularization parameter. Alternatively, one may invert a constrained likelihood ratio test with the *L*_0_-constraint (Zhu et al., 2020). Yet, the inherent bias due to regularization persists in the finite sample regime even after debiasing.

To construct a confidence interval for parameter *β*_*l*_, we apply the constrained *L*_0_-norm regression (Shen et al., 2012) to select variables excluding variable *X*_*l*_ while treating other regression parameters as a nuisance, where the truncated *L*_1_-penalty function (TLP) constraint approximates the *L*_0_-constraint for computation. Towards this end, we apply the data-flush Monte-Carlo inference method based on (2.1) for a confidence interval to generate synthetic samples to estimate the distribution of an asymptotic pivotal quantity $T=\left({\hat{\beta }}_{l}-\beta_{l}\right)/SE\left({\hat{\beta }}_{l}\right)$ (Zhu et al., 2020), where $SE\left({\hat{\beta }}_{l}\right)$ is the standard error of the constrained *L*_0_-norm regression (CTLP) estimate ${\hat{\beta }}_{l}$.

To replicates ${\left\{X_{i},Y_{i}\right\}}_{i=1}^{n}$ for inference, we apply (2.1), where *e*_*ij*_ is independently sampled from the *Laplace*(0, 1*/ε*) distribution and *ε* = 0.01, Then, $Y_{ij}^{*}=\hat{\mu }\left(X_{i}\right)+\varepsilon_{ij}^{*}$ satisfies *ε*-differential privacy for any *j*, where $\varepsilon_{ij}^{*}=R^{-1}(G\left(\left(U_{i}+e_{ij}\right)\right)$ in (2.1) and $\hat{\mu }\left(X_{i}\right)=\sum_{l=1}^{p}{\hat{\beta }}_{l}X_{il}$ and ${\hat{\sigma }}^{2}$ are the fitted value and the standard estimate of *σ*^2^ based on a holdout sample that is independent of the inference sample, *R* is the CDF of $N\left(0,\;{\hat{\sigma }}^{2}\right)$, and *G* is the CDF of *U*_*i*_ +*e*_*ij*_ with *U*_*i*_ following the Uniform[0, 1] distribution.

We perform simulations with the true parameters *β*_1_ = *β*_2_ = *β*_3_ = 1 and *β*_*j*_ = 0 otherwise, with *σ* = 0.5 and *ρ* = 0.5. Then, we apply (2.1) with *m* = *D/n* and *D* = 10*p* to construct a 95% confidence interval for each *β*_*j*_ based on CTLP. The results for *β*_1_ and *β*_4_ are representative and are presented. Specifically, we use the glmtlp package in R to compute the CTLP estimate ${\hat{\beta }}_{l}$ and the default ${\hat{\sigma }}^{2}$ there.

Table 2 shows that the empirical coverage probability for *β*_1_ and *β*_4_ are close to the nominal level 95% in each scenario. The discrepancy between the empirical converge and its target 95% is because the asymptotic pivotal may suffer from the bias in the finite-sample situation. Overall, the data-flush Monte-Carlo inference scheme yields a credible confidence interval for a non-smooth problem involving model selection.

## 4. American Community Survey data analysis

This section applies the data-flush scheme (2.2) to the 2019 American Community Survey (ACS) Data. Notice that, the existing literature in privacy has not thoroughly depicted low-error-high-privacy differentially private methods for complex sample surveys such as the ACS (Reiter, 2019). We show that data generated by data flush is valid for statistical inference while simultaneously guaranteeing differential privacy. In particular, we demonstrate that confidence intervals constructed upon perturbed copies of raw data are close to those on perturbed copies of privatized data. In other words, the data-flush scheme can simultaneously achieve two disparate objectives: differential privacy and statistical inference.

The American Community Survey collects demographic data from 3.24 million persons nation-wide, roughly 1% of the population in the Year 2019 (Ruggles et al., 2021). Statistical analysis of survey data has a long history. Muralidhar and Sarathy, 2003 provided a theoretical basis for data perturbation with a definition of disclosure risk requirement. Raghunathan et al., 2003 and Reiter, 2005 proposed to use multiple imputation to limit the disclosure risk of microdata. Woodcock and Benedetto, 2009 applied a transformation to maximize data utility while minimizing incremental disclosure risk. Jiang et al., 2021 proposed a perturbation method with a masking component to preserve inferential conclusions such as confidence intervals. While most of the above methods aim at limiting the data disclosure risk, they are not designed for differential privacy and are not able to preserve distributions for most data types.

Alternatively, an investigator can apply data flush to privatize survey data like ACS data without incurring information loss when the data-flush scheme preserves the distribution of raw data. For the ACS dataset, we use (2.2) for privatization while applying the data-flush Monte-Carlo inference method to both the raw and privatized data. For an illustration, we make a pairwise comparison of two confidence intervals before and after privatization for coefficients of weighted regression.

In particular, we investigate the impact of privatization by (2.2) on the statistical accuracy of regression analysis of the total personal income on 16 covariates, including an individual’s age (AGE), geographical region (REGION), the population of the residential metro/micro area (METPOP10, the logarithm of METPOP10 to be used), metropolitan status (METRO), mortgage status (MORTGAGE), sex (SEX), marital status (MARST), race (RACE), ethnicity (HISPAN), ability to speak English (SPEAKING), health insurance coverage (HCOVANY), educational attainment (EDUCD), employment status (EMPSTAT), occupation (OCC), migration status (MIGRATE1), and veteran status (VETSTAT). For our analysis, we select individuals with a positive total pre-tax personal income from all sources during the 12 months precedent to the survey. This preprocessing renders a sample of 2,389,971 individuals. See the Appendix for more specific details regarding preprocessing. The data types, as well as the number of levels for nominal variables, are summarized in Table 3. Then, we regress the logarithm of total personal income on these 16 covariates using the person weight (PERWT) as the weights for regression. A confidence interval (CI) for each regression coefficient is constructed accordingly before and after privatization.

**Figure 2.**
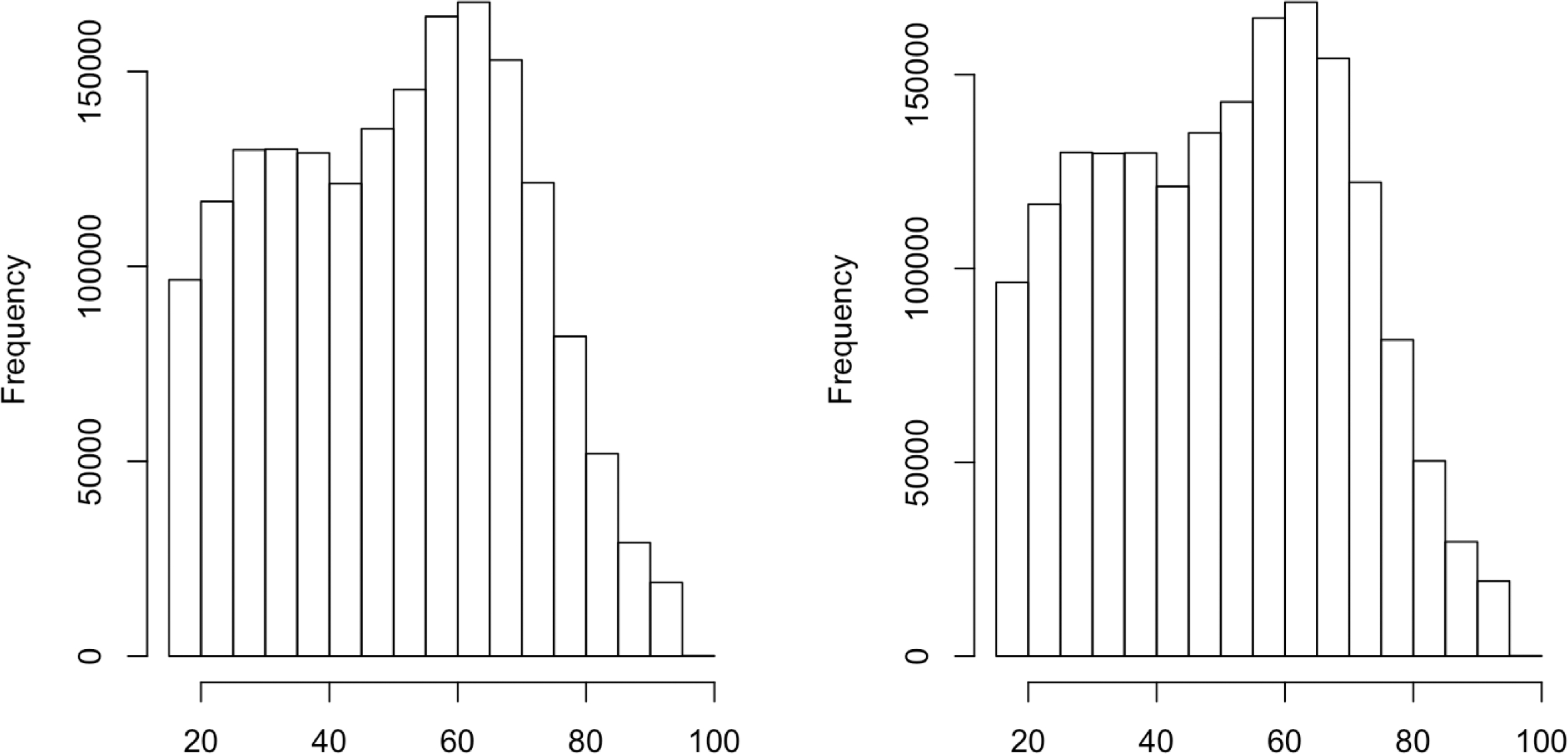
Histogram of the AGE variable in the ACS data before and after privatization.

To satisfy *ε*-differential privacy, we apply (2.2) with *e*_*ij*_ following a *Laplace*(0, 17*/ε*) distribution to preserve the joint distribution of 16 covariates and 1 response variable across common data types. In this fashion, privatization protects each individual’s information. To illustrate this point, we scrutinize the histogram of the variable AGE before and after privatization in Figure 2, which suggests that little distributional difference is evident. Note that the two histograms before and after privatization are nearly identical, with the mean (standard deviation) being 50.80(19.17) and 50.82(19.17), respectively. Moreover, we randomly choose two categorical variables, namely employment status (EMPSTAT) and migration status (MIGRATE1), to examine the joint distribution before and after privatization, which are the 13th and 15th variables out of 17 variables in the sequential privatization process through (2.2). As suggested by Table 4, the data flush scheme preserves the joint distribution quite well after privatization, particularly for the two-way associations, except for one cell (States-abroad, Non-labor) with small counts. In conclusion, the distribution preservation property of data flush ascertains the validity of downstream statistical inference while protecting data privacy.

**Figure 3.**
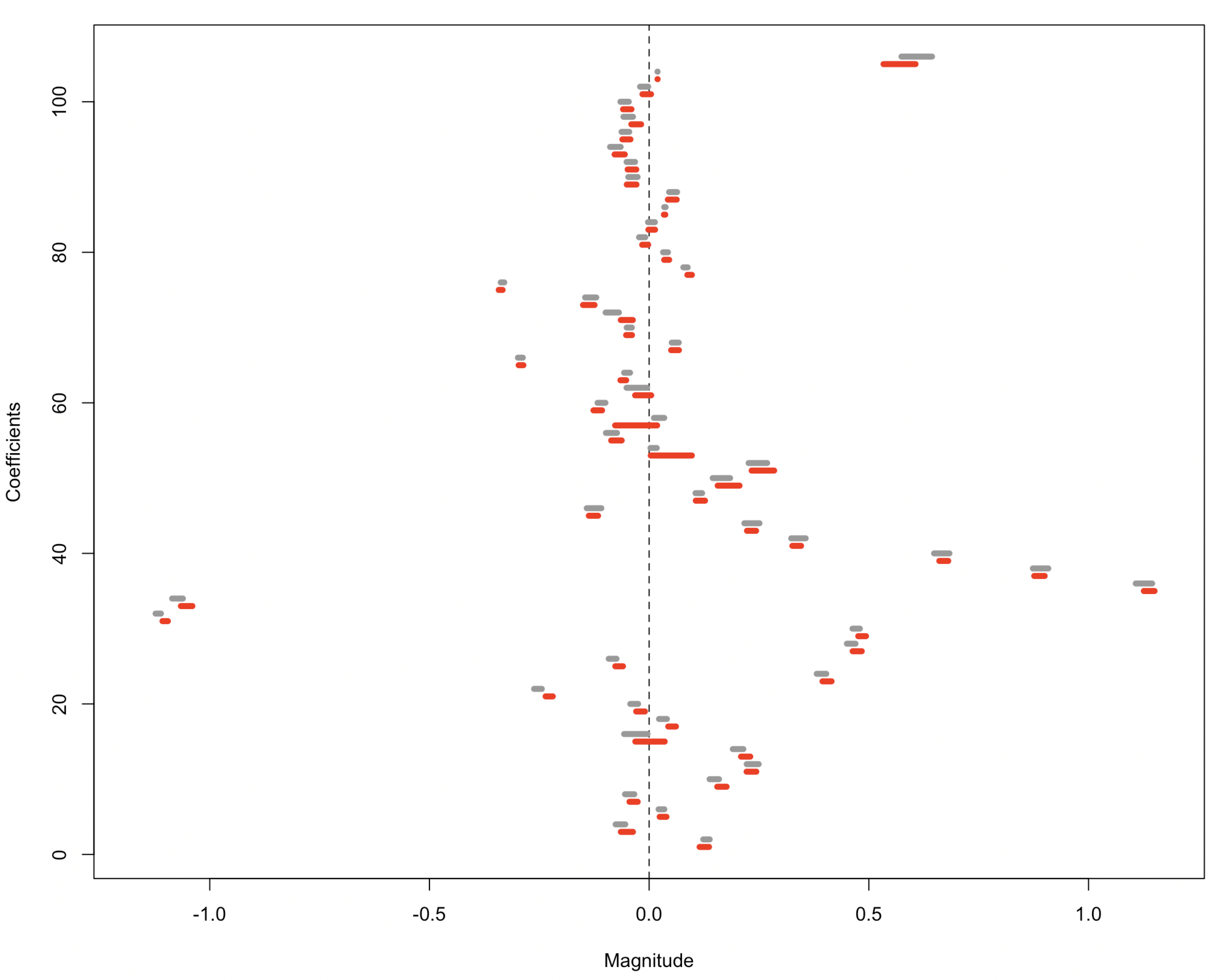
Confidence intervals of regression coefficients based on raw data and privatized data, represented by gray and red lines and constructed using the data-flush scheme in Section 3. Regressors from the top to the bottom are the intercept (shifted to the left by 8 units for better visualization), AGE, REGION (8 dummy variables), METPOP10, METRO (2 dummy variables), MORTGAGE (2 dummy variables), SEX, MARST (5 dummy variables), RACE (5 dummy variables), HISPAN, SPEAKENG (2 dummy variables), HCOVANY, EDUCD (6 dummy variables), EMPSTAT (2 dummy variables), OCC (12 dummy variables), MIGRATE (2 dummy variables), and VETSTAT. The confidence intervals based on raw data are comparable with those after privatization in terms of the signs of interval centers and lengths.

We apply the data-flush Monte-Carlo method to construct confidence intervals for raw and privatized data. In particular, for each replication, we only perturb the linear regression residuals and follow the high-dimensional regression example in Section 3. As indicated by Figure 3, the data-flush scheme (2.2) preserves the target distribution of raw data and hence yields nearly identical confidence intervals except for several ones with shifting centers.

Privacy loss usually occurs for high-dimensional data, which is an inherent challenge for any method in differential privacy. In particular, to maintain the same accuracy level, the overall level of privacy protection for each variable tends to decay as the number of variables increases. In our situation, the overall level of privacy protection, defined by the privacy factor *ε*, is 1 for *ε*-differential privacy, which requires a stricter level of privacy protection 1/17 for each of the 17 variables. It is equivalent to that each variable requires independent *Laplace*(0, 17*/ε*), where the noise variance greatly exceeds the ranges of many variables in the ACS data, especially for binary dummy variables.

## 5. Discussion

Data perturbation has its great potential as an effective tool for replicating a sample, which can apply to data security, statistical inference, data integration, among others. The fundamental principle, distribution preservation for data perturbation, that we described in this article allows users to design data perturbation schemes, such as data flush, to satisfy task-specific requirements, as we showcase for statistical inference with differential private data in Section 4. On this ground, synthetic data generated by such a scheme yields statistically valid analysis and high predictive accuracy of a machine learning task.

Several future directions of research include a more flexible model-based estimation (e.g., one including both parametric and empirical components) for high-dimensional target distributions and a compatible data perturbation scheme, as well as generalizations to independent but non-identically distributed data, time-series data, and unstructured data.

## Figures and Tables

**Table 1.** Private Poisson regression with a privacy factor *ε* = 1 using raw data, data privatized by data-flush in (2.2), and data privatized by GEM (Liu, Vietri, & Wu, 2021). Kullback-Leibler divergence (KL) and root mean square error (RMSE) for regression coefficients (with the standard error in parenthesis), together with privatization time (Time, in seconds) are presented based on 200 replications. Here σ is the standard deviation of each covariate before discretization (a step required by GEM), and NA indicates that an algorithm fails to converge within two days.

	σ = 1	σ = 10	σ = 100

KL			

Raw data	0.001 (0.001)	0.001 (0.001)	0.001 (0.001)
GEM	0.140 (0.126)	NA	NA
Data-flush	0.005 (0.003)	0.005 (0.003)	0.005 (0.004)

RMSE			

Raw data	0.040 (0.014)	0.005 (0.002)	0.001 (0.0002)
GEM	0.273 (0.108)	NA	NA
Data-flush	0.090 (0.033)	0.013 (0.005)	0.001 (0.0005)

Time			

GEM	423.25	NA	NA
Data-flush	0.35	0.34	0.33

**Table 2.** Empirical coverage probability (Coverage %) of a 95% confidence interval for *β*_1_ and *β*_4_ based on CTLP over 500 simulations in (3.1), where *p*, *n*, *D* represent the number of predictors, the sample size, and the Monte Carlo size, respectively.

	*P*	*n*	*D*	% Coverage

*β*_1_	50	100	1000	92.4
*β*_1_	200	100	2000	93.0
*β*_1_	500	100	5000	94.6

*β*_4_	50	100	1000	95.4
*β*_4_	200	100	2000	93.6
*β*_4_	500	100	5000	92.0

**Table 3.** Summary statistics for variables used in the ACS analysis, including variable’s names (Name), types (Type), the number of levels for nominal variables (# Level), as well as the mean (Mean) and standard deviation (Standard deviation). Here NA means “Not applicable”.

Name	Type	# Level	Mean (Standard deviation)

AGE	empirical	NA	50.80 (19.17)
REGION	nominal	9	NA
METPOP10	empirical	NA	3.30 × 10^6^ (5.00 × 10^6^)
METRO	nominal	5	NA
MORTGAGE	nominal	3	NA
SEX	binary	NA	0.50 (0.50)
MARST	nominal	6	NA
RACE	nominal	6	NA
HISPAN	binary	NA	0.12 (0.32)
SPEAKENG	nominal	3	NA
HCOVANY	binary	NA	0.93 (0.26)
EDUCD	nominal	7	NA
EMPSTAT	nominal	3	NA
OCC	nominal	13	NA
MIGRATE1	nominal	3	NA
VETSTAT	binary	NA	0.08 (0.28)
INCTOT	continuous	NA	51365.44 (69097.25)

**Table 4.** Joint distribution between employment status (EMPSTAT) and migration status (MIGRATE1) before and after privatization, where each cell in the contingency table indicates the number of individuals in the release sample before (after) privatization. For MIGRATE1, “House”, “State”, and “States-Abroad” indicate staying in the same house, moving within a state, and moving between states or abroad; for EMPSTAT, “Employed”, “NA/Unemployed”, and “Non-labor” mean that an individual is employed, unemployed or not applicable, and not in the labor force, respectively.

	EMPSTAT			
MIGRATE1	Employed	NA/Unemployed	Non-labor	Total

House	996078 (1012469)	31207 (31163)	542095 (574287)	1569380 (1617919)
State	120963 (100515)	5697 (4652)	48451 (32242)	175111 (137409)
States-abroad	32120 (31436)	2072 (2230)	13796 (3485)	47988 (37151)

Total	1149161 (1144420)	38976 (38045)	604342 (610014)	1792479 (1792479)

## Appendix A. Proof of Lemma 1

By construction, $\left(U_{1}^{\left(1\right)},\cdots ,U_{n}^{\left(1\right)}\right)$ follows the uniform distribution and retains the ranks of $\left(Z_{1}^{\left(1\right)},\cdots ,Z_{n}^{\left(1\right)}\right)$. Then, the Spearman’s rank correlation $\rho \left({\left\{Z_{i}^{\left(1\right)}\right\}}_{i=1}^{n},{\left\{U_{i}^{\left(1\right)}\right\}}_{i=1}^{n}\right)=1$. It follows from the strictly increasing property of *H*^(1)^ that $\rho \left({\left\{Z_{i}^{\left(1\right)}\right\}}_{i=1}^{n},{\left\{Z_{ij}^{(1)*}\right\}}_{i=1}^{n}\right)=1$ when *e*_*ij*_ = 0 for any fixed *j*. By continuity, $\rho \left({\left\{Z_{i}^{\left(1\right)}\right\}}_{i=1}^{n},{\left\{Z_{ij}^{(1)*}\right\}}_{i=1}^{n}\right)\to 1$ as *e*_*ij*_ → 0 in probability. This completes the proof.

## Appendix B. Simulation Comparison for Poisson Regression

To compare with the method of GEM (Liu, Vietri, & Wu, 2021), we generate a sample of paired data ${\left(X_{i},Y_{i}\right)}_{i=1}^{n}$, where **X**_*i*_ and *Y*_*i*_ need to be discrete to accommodate the requirement for GEM. First, we sample (**X**_1_,···,**X**_*n*_) from a 5-dimensional normal distribution *N*(**0**, **Σ**), where the off-diagonal and diagonal values of the covariance matrix **Σ** are 0.7*σ*^2^ and *σ*^2^, and *σ* = 1, 10, 100. Then, we discretize them by rounding each component of **X**_*i*_ to the smallest integer above its value. The average numbers of distinct values for (**X**_1_,···,**X**_*n*_) are 8, 64, and 465, respectively for *σ* = 1, 10, and 100. This discretization allows us to evaluate the performance of each method under an unknown true distribution. Second, we generate a Poisson response *Y*_*i*_ with mean $\exp\left(X_{i}^{\prime }\beta \right)$; *i* = 1, · · ·,*n*, where $\beta =\left(\frac{1}{5\sigma },\ldots ,\frac{1}{5\sigma }\right)$ yields a reasonable range of *Y*_*i*_.

For data flush, we set the privacy factor to be *ε* = 1 to ensure strict protection under *ε*-differential privacy. To apply (2.2), we randomly select 25% of ${\left(X_{i},Y_{i}\right)}_{i=1}^{n}$ as a holdout sample to construct a smoothed empirical CDF and use the remaining sample for privatization. For GEM, we let $(\varepsilon ,\delta )=\left(1,\frac{1}{n^{2}}\right)$ for (*ε*, *δ*)-differential privacy. Note that (*ε*, *δ*)-differential privacy with *δ* = 0 reduces to *ε*-differential privacy. GEM intends to preserve *W* three-way interactions, where we choose *W* = 5 out of 20 possible three-way interactions from 6 variables (*Y*_*i*_ and components of **X**_*i*_), with *W* denoting the number of interactions to consider. Then, we apply the GEM algorithm^1^ in Liu, Vietri, and Wu, 2021 to privatize ${\left(X_{i},Y_{i}\right)}_{i=1}^{n}$ of *n* = 2500 using the default values with the number of iterations *T* = 10.^2^

Given privatized data, we obtain estimated regression coefficient vector $\hat{\beta }$ in Poisson regression and evaluate predictive performance by the Kullback-Leibler divergence and parameter estimation by the root mean square error between the estimated and true regression coefficients ***β***. As a reference, we also report simulation results on the non-private data ${\left(X_{i},Y_{i}\right)}_{i=1}^{n}$.

## Appendix C. Proof of Theorem 1

By the definition of a pivotal quantity, $T\left(\theta_{1},{\hat{\theta }}_{1}\right)$ has the same distribution as $T\left(\theta_{2},{\hat{\theta }}_{2}\right)$ when ${\hat{\theta }}_{j}=\hat{\theta }\left(Z^{\left(j\right)}\right)$ is obtained via the same statistical procedure, where ***Z***^(*j*)^ follows *F*(*θ*_*j*_); *j* = 1, 2. Let *θ*_1_ = *θ* and ${\hat{\theta }}_{1}=\hat{\theta }(Z)$. Let $\theta_{2}=\hat{\theta }(Z)$ and ${\hat{\theta }}_{2}=\hat{\theta }\left(Z^{*}\right)$. Note that ***Z***^∗^ given ***Z*** follows $F(\hat{\theta })$ while ***Z*** follows *F*(*θ*). Therefore, the conditional distribution of $T^{*}=T\left(\theta_{2},{\hat{\theta }}_{2}\right)$ given ***Z*** remains the same as the unconditional distribution of $T=T\left(\theta_{1},{\hat{\theta }}_{1}\right)$ for any ***Z***. This completes the proof.

## Appendix D. ACS Data Preprocessing

We preprocess the 2019 American Community Survey data, available at https://usa.ipums.org. For variable METRO, we combine all other levels exceeding level 2 with level 2 to form a new level 2 to indicate “In metropolitan area”. For variable MORTGAGE, we merge all levels above level 3 into level 3 to indicate “Yes, have or will have mortgage”. For the RACE variable, we merge levels 4 (“Chinese”) and 5 (“Japanese”) with level 6 to represent “Other Asian or Pacific Islander” and merge level 9 (“Three or more major races”) into level 8 (“Two major races”) to represent (“More than one major race”). For variable HISPAN, we merge levels from 1 to 4 into level 1 to represent “Hispanic” as there are no individuals in the data reporting 9 (“Not Reported”). For variable SPEAKING, we merge levels 4 and 5 into level 6 to indicate (“Speak English, but not only English”). For variable EDUCD, we merge levels 0 to 2 into 0 to represent “No school (completed),” levels from 10 to 61 into 1 to indicate “Nursery school to grade 12”, and levels from 62 to 64 into 2 to represent “High school graduate, GED, or alternative credential”, levels from 65 to 100 into 3 for “Some college”, level 101 into 4 for “Bachelor’s degree”), level 114 into 5 for “Master degree”, and levels 115 and 116 into level 6 for “Professional degree beyond a bachelor’s degree or Doctoral degree”. No other levels are available for EDUCD. For variable OCC, we merge occupations based on the 13 subcategories provided at https://usa.ipums.org/usa/volii/occ2018.shtml, including “Not Applicable”, “Management, Business, and Financial Occupations”, “Computer, Engineering, and Science Occupations”, “Education, Legal, Community Service, Arts, and Media Occupations”, “Healthcare Practitioners and Technical Occupations”, “Service Occupations”, “Sales and Related Occupations”, “Office and Administrative Support Occupations”, “Farming, Fishing, and Forestry Occupations”, “Construction and Extraction Occupations”, “Installation, Maintenance, and Repair Occupations”, “Production Occupations”, and “Transportation and Material Moving Occupations”. For EMPSTAT, we merge “N/A” with “Unemployed”, and for MIGRATE1, we merge “Moved between states” and “Abroad one year ago”. For VETSTAT, we merge “N/A” with “Not a veteran”. For METPOP10 and INCTOT, we take the logarithmic transformation before fitting regression to deal with the long-tail distribution. The remaining variables are intact.

## Appendix E. Implementation Details of the ACS Data

After sampling 25% of the ACS data as the holdout sample and leaving the other 75% as the to-be-privatized sample to be released, we apply (2.2) to 16 covariates in addition to the response sequentially following the order and variable types listed in Table 3.

To achieve privatization, we estimate the conditional distribution of each variable given all the previous variables via a corresponding generalized linear model. First, we prioritize AGE using the marginal empirical distribution of AGE based on the holdout sample. Second, we privatize REGION by fitting multinomial logistic regression of REGION on AGE in the holdout sample to estimate the corresponding parameters and then compute the probability of each REGION in the to-be-privatized data given the privatized AGE as the new covariate. This privatization process continues with the remaining variables following the sequential order in Table 3 and using an estimated conditional distribution on the holdout sample by logistic regression, multinomial logistic regression, and linear regression for binary, nominal, and normally-distributed data such as log(INCTOT). Note that we privatize METPOP10 by the conditional distribution of METPOP10 given REGION without using AGE. This assumption appears sensible given that the sample correlation is −0.036 between the area and the participant’s age.

The data-flush scheme in (2.2) generates *D* = 500 conditionally independent samples with noise independently following *Laplace*(0, 0.2), followed by the three steps described in Section 3 with a confidence level of 95%.
